# Supplementary material for: Copolymerization of Norbornene and Styrene with Anilinonaphthoquinone-Ligated Nickel Complexes
Source: Polymers (Basel). 2019 Jun 28;11(7):1100. doi: 10.3390/polym11071100 (PMC6680643; doi:10.3390/polym11071100)
Supplement: Supplementary file 1 [file polymers-11-01100-s001.pdf]

Supporting Information

Copolymerization of Norbornene and Styrene with Anilinonaphthaquinone-ligated Nickel Complexes

Synthesis of Anilinonaphthoquinone ligand 1c: The ligand was synthesized by applying the literature procedure [39]. First, aniline (8.6 mmol) was added drop wise into a solution of 2-hydroxy-1,4-naphthoquione (1.50 g, 8.6 mmol) in *m*-cresol (30 mL) in the presence of trifluoroacetic acid (0.20 mL, 2.69 mmol) as a catalyst. The mixture was heated with stirring at 100 °C, for 4 h and then poured into 900 mL of 5 wt.-% aqueous sodium hydroxide. The precipitate formed was filtered, washed with water, and dried under a vacuum at 80 °C for 6 h. The ligand powder was purified by recrystallization using acetic acid. The yield was 1.37 g (5.43 mmol, 63%).

^{1}H NMR ($CDCl_3$, 500 MHz): δ = 8.13 (dt, 2H), 7.78 (dt, 1H), 7.69(dt, 1H), 7.58(br, 1H), 7.44(dt, 2H), 7.30 (d, 2H), 7.23 (d, 1H), 6.44 (s, 1H),

^{13}C NMR ($CDCl_3$, 500 MHz): δ = 184.1, 181.7, 144.9, 137.1, 134.9, 133.2, 132.3, 130.4, 129.7, 126.5, 125.9, 125.5, 122.6, 103.3.

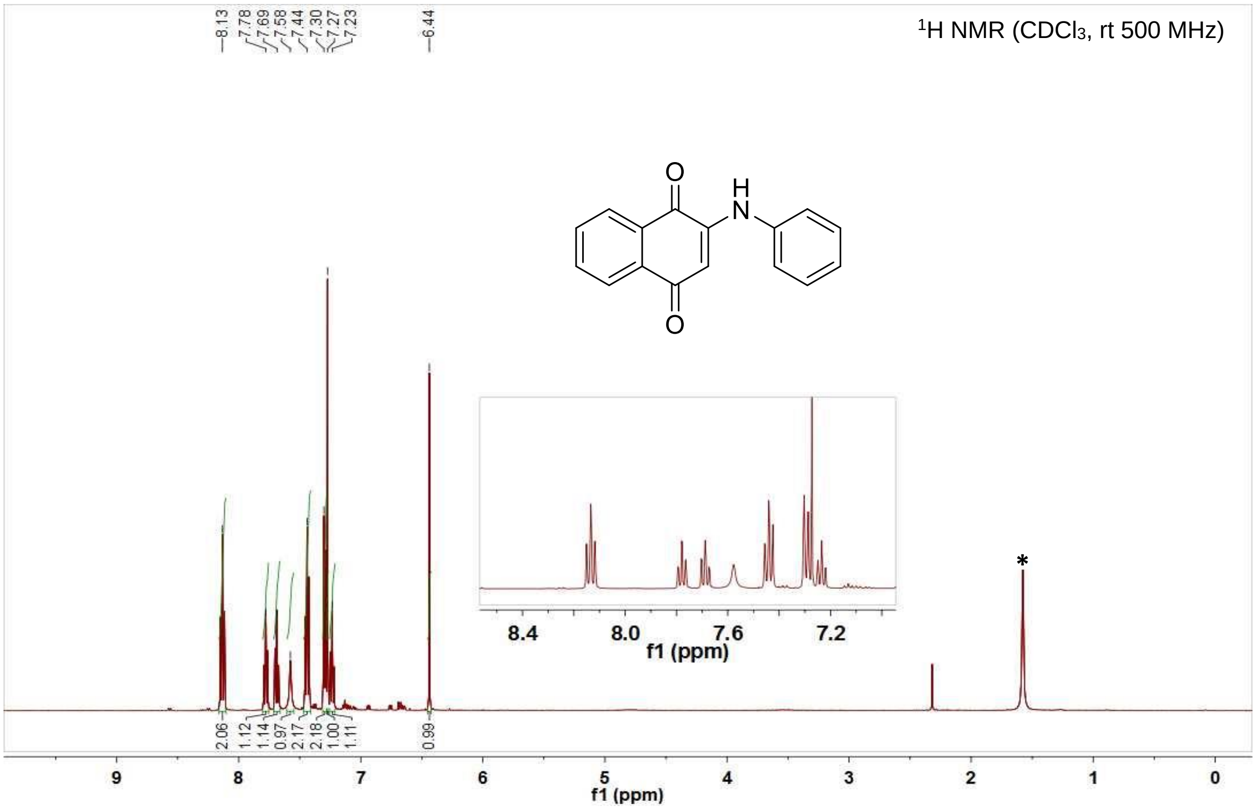

Figure S1. ^{1}H NMR spectrum of ligand 1c (*H_2O).

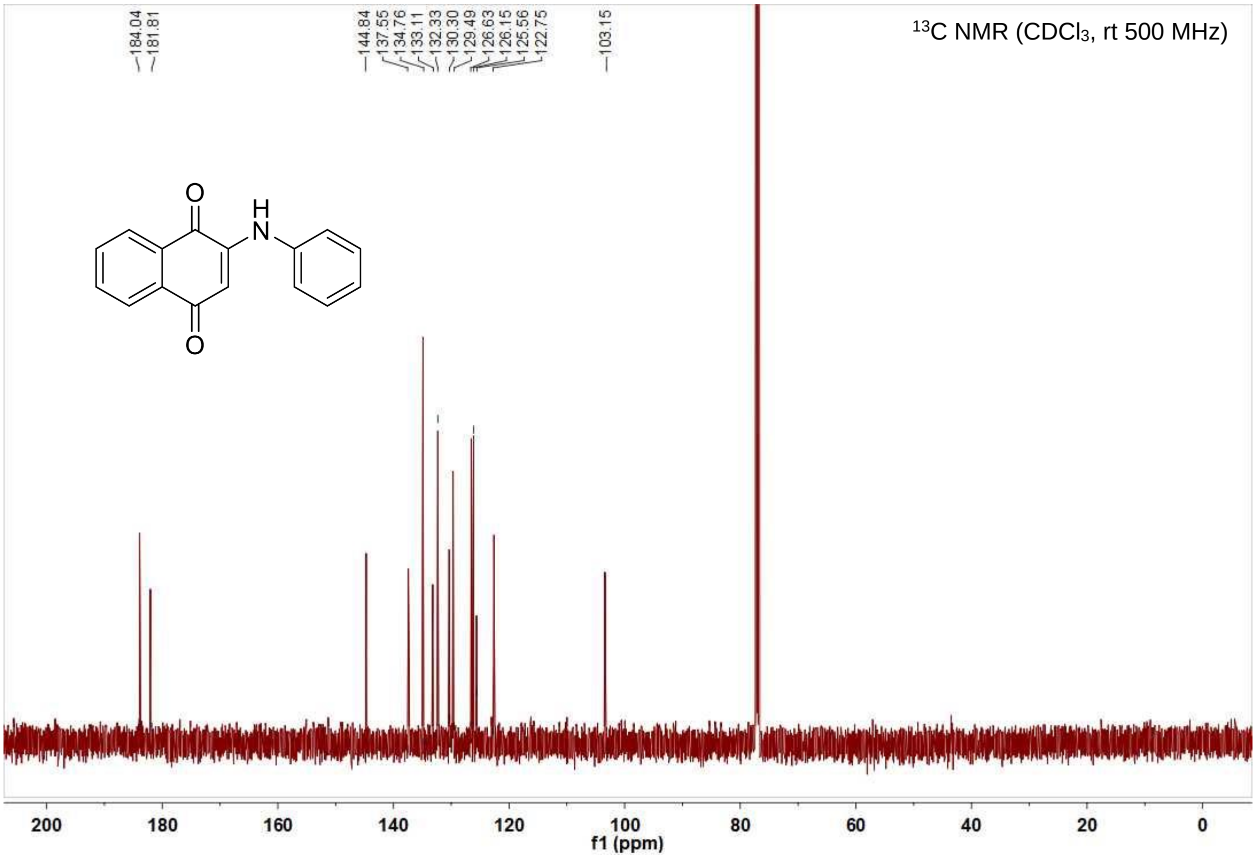

Figure S2. ^{13}C NMR spectrum of ligand1c.

Synthesis of Complex 1c: The nickel complex was synthesized by applying the literature procedure [39]. The ligand (1.01 g, 3.03 mmol) in THF (20 mL) was slowly added through a dropping funnel into a reactor containing a slurry of NaH (0.08 g, 3.31 mmol) in THF (10 mL) cooled using an ice-water bath at 0 °C, and the resultant slurry was stirred for 3 h at room temperature. The slurry thus obtained was filtered off under a nitrogen atmosphere. The residue was washed with THF, and dried under a vacuum at room temperature for 6 h. The sodium salt of the ligand contained 1 eq. of THF. The sodium salt of the ligand (0.72 g, 1.68 mmol) and trans-[Ni(PPh_3)$_2$PhCl] (1.18 g, 1.68 mmol) [1], which was prepared according to the literature [39], were mixed in a Schlenk tube with THF (10 mL) at room temperature and stirred for 1 d. The reaction mixture was filtered off under a nitrogen atmosphere, and the filtrate was evaporated to dryness under a vacuum. The solid thus obtained was purified with a mixture of THF/hexane in a 1/5 volume ratio. The powder was determined by ^{1}H and ^{31}P NMR. The yield was 0.37 g (0.57 mmol, 33%).

^{1}H NMR (C_6D_6, 500 MHz): δ = 8.15 (d, 1H), 7.65 (br, 2H), 7.35(m, 7H), 6.99(m, 19H), 6.4 (s, 1H), 3.5 (THF).

^{31}P NMR (C_6D_6, 202 MHz): δ = 28.96.

Elemental analysis calculated: C, 74.33; H, 4.68, N, 2.17. Found: C, 74.19; H, 5.01; N, 2.23.

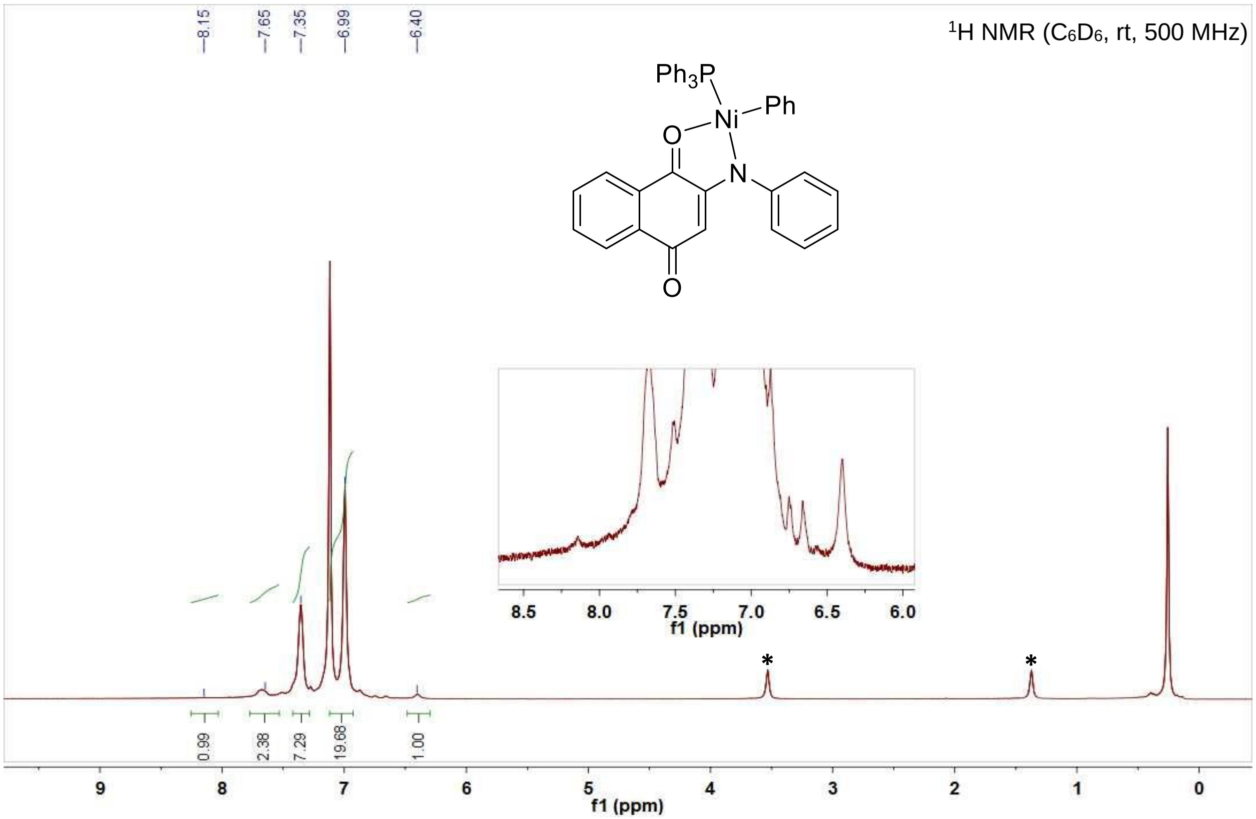

Figure S3. ^{1}H NMR spectrum of complex **1c** (* THF).

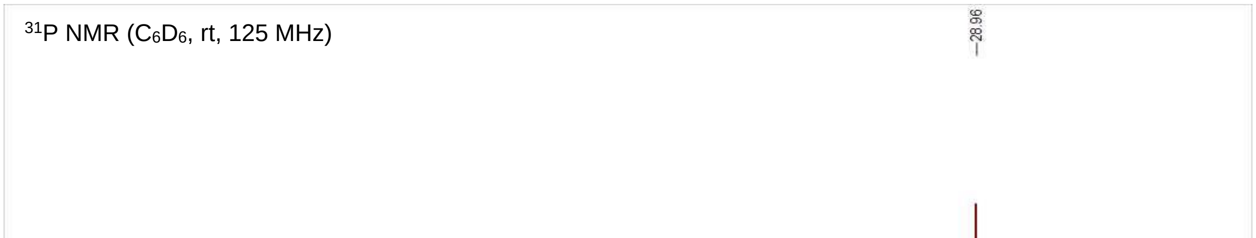

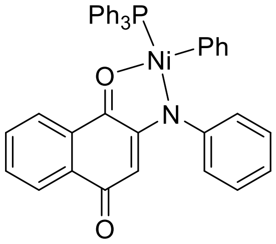

Figure S4. ^{31}P NMR spectrum of complex **1c**.

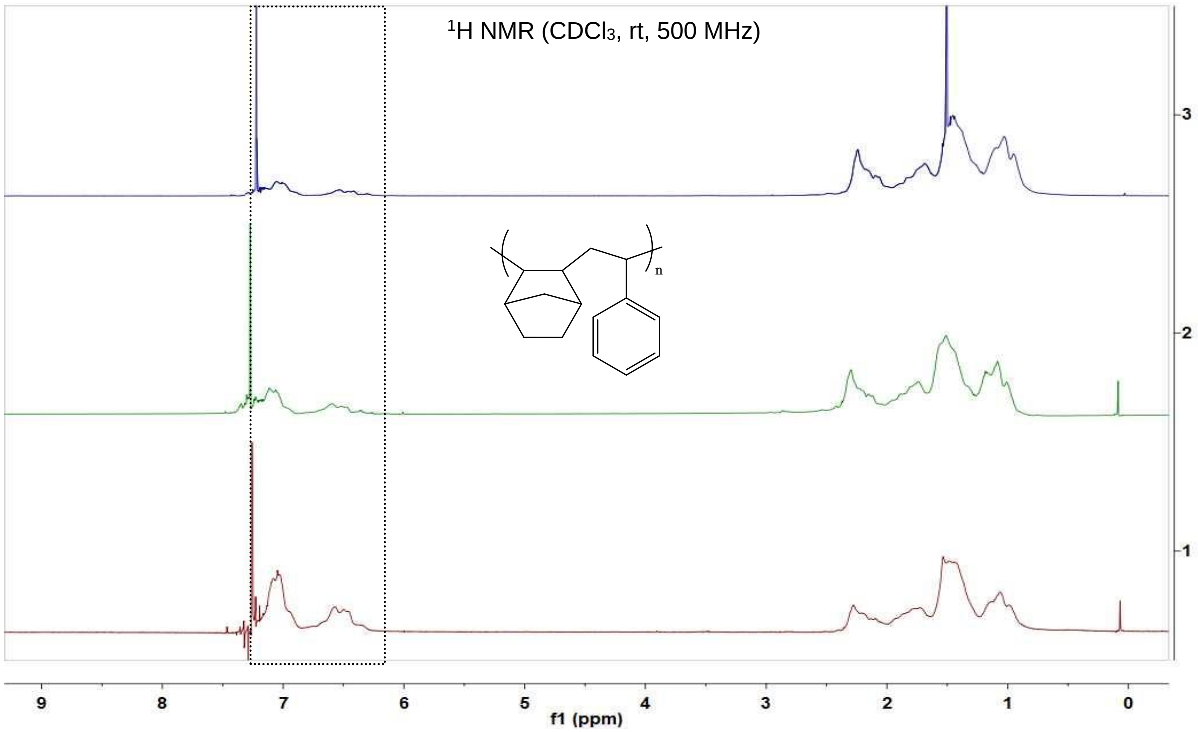

Figure S5. ^{1}H NMR spectra of N/S copolymers obtained by (**1**) Run15, (**2**) Run 9, and (**3**) Run 3 [$CDCl_3$, 500 MHz].

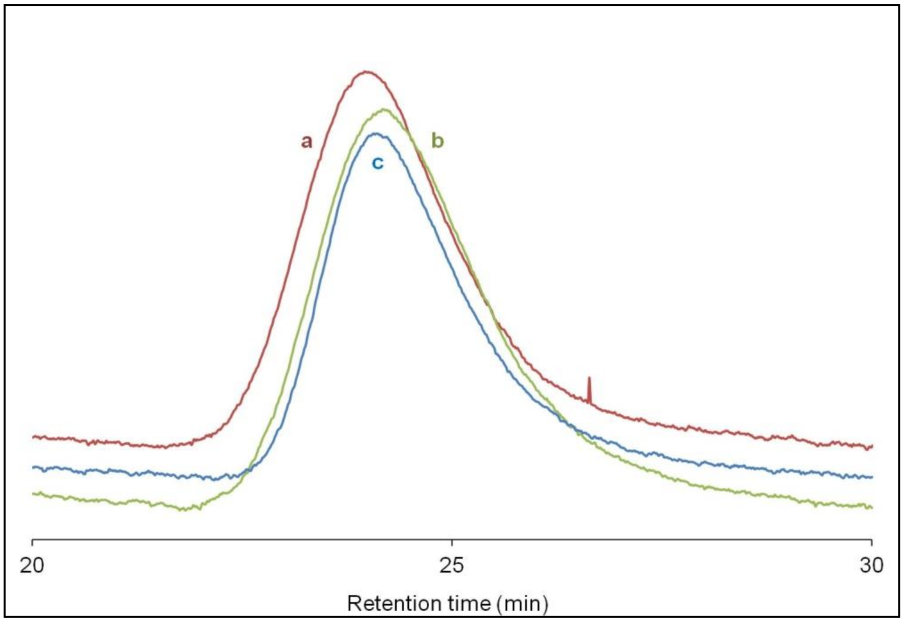

Figure S6. GPC traces N/S copolymers: **a**, Run 7; **b**, Run 8; **c**, Run 9.

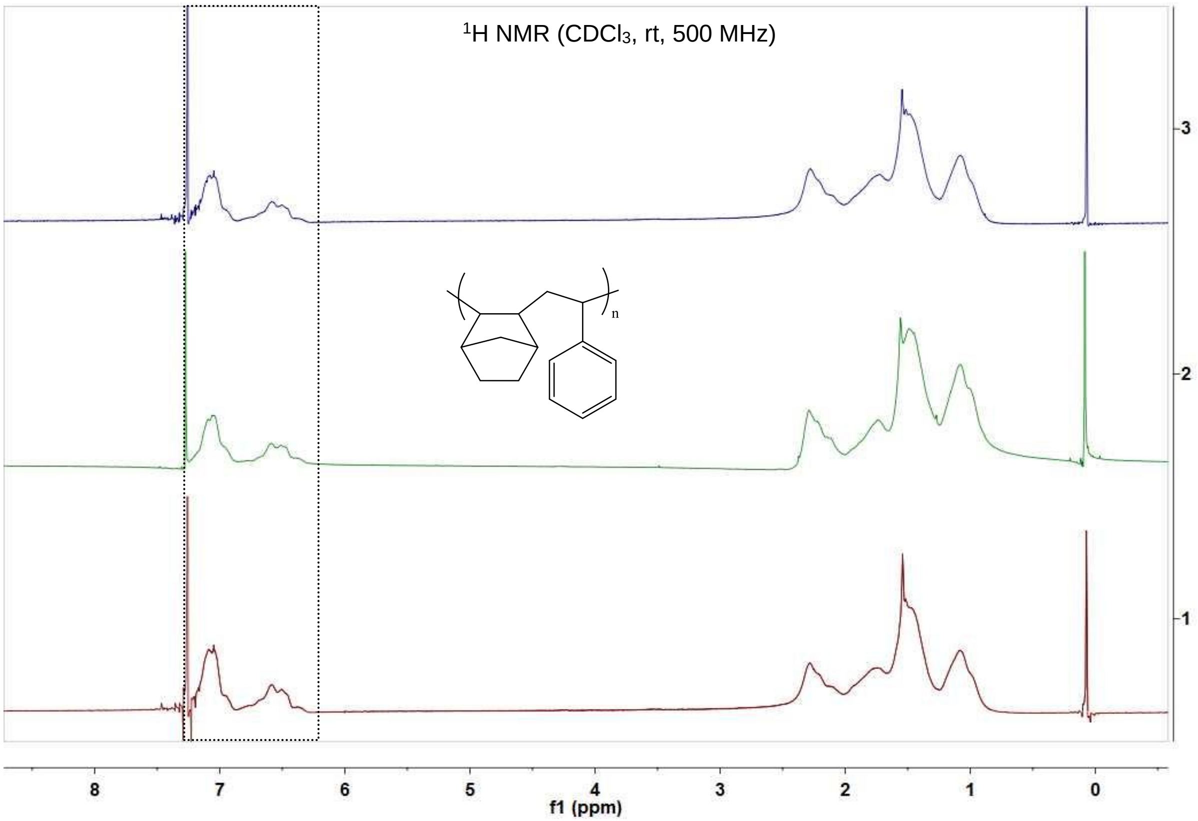

Figure S7. ^{1}H NMR spectra of N/S copolymers obtained by (**1**) Run 25, (**2**) Run 22, and (**3**) Run 19 [$CDCl_3$, 500 MHz].

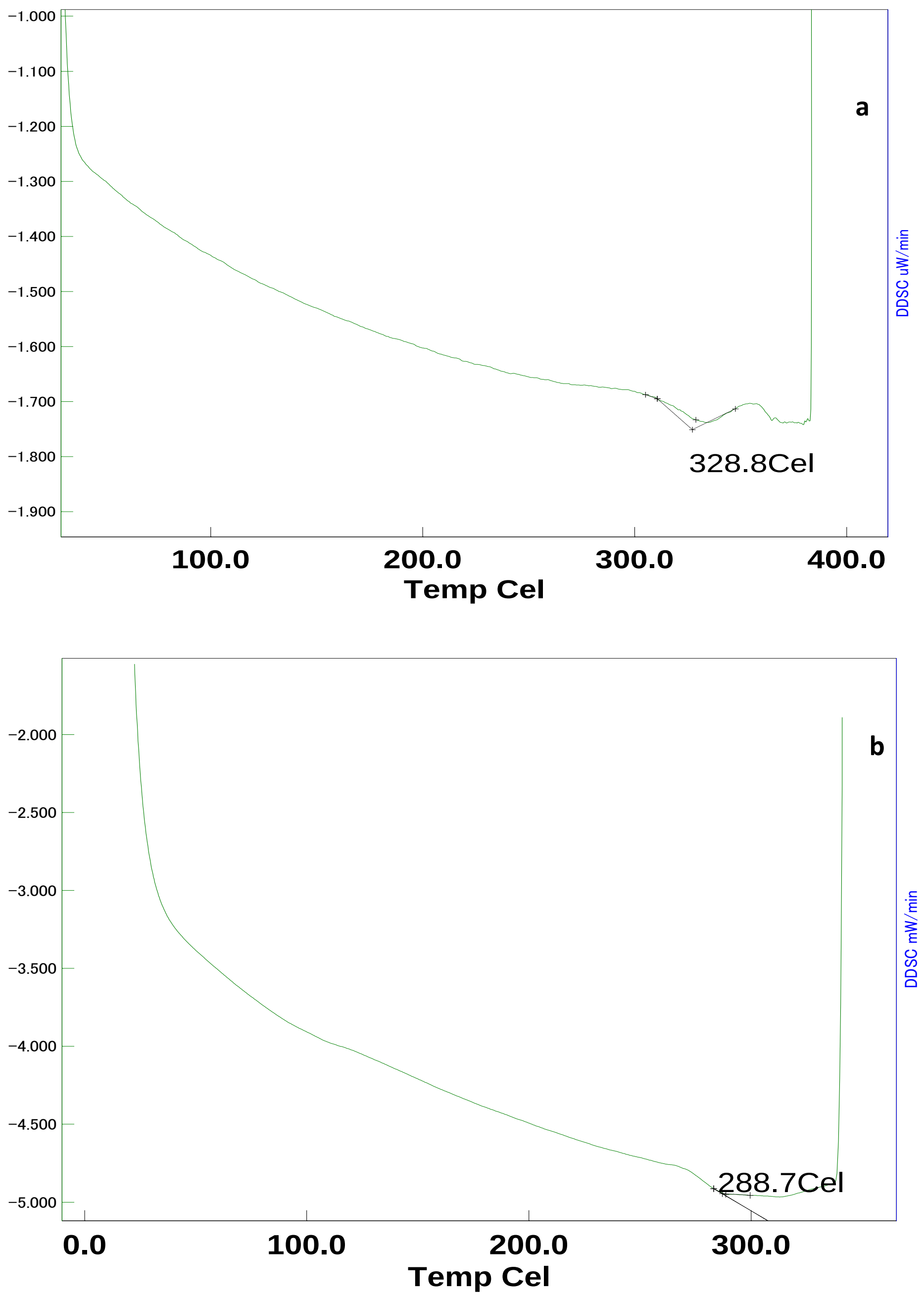

Figure S8. DSC curves of N/S copolymers obtained by (**a**) Run 6 and (**b**) Run 17.

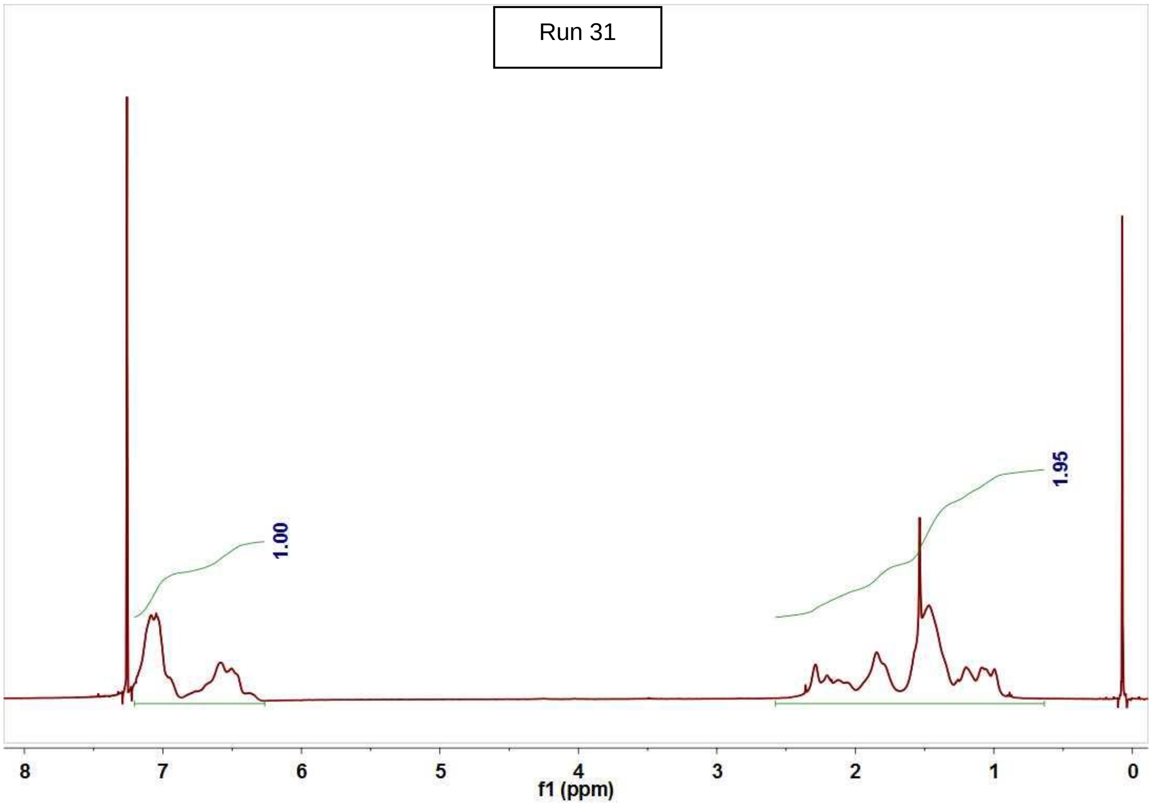

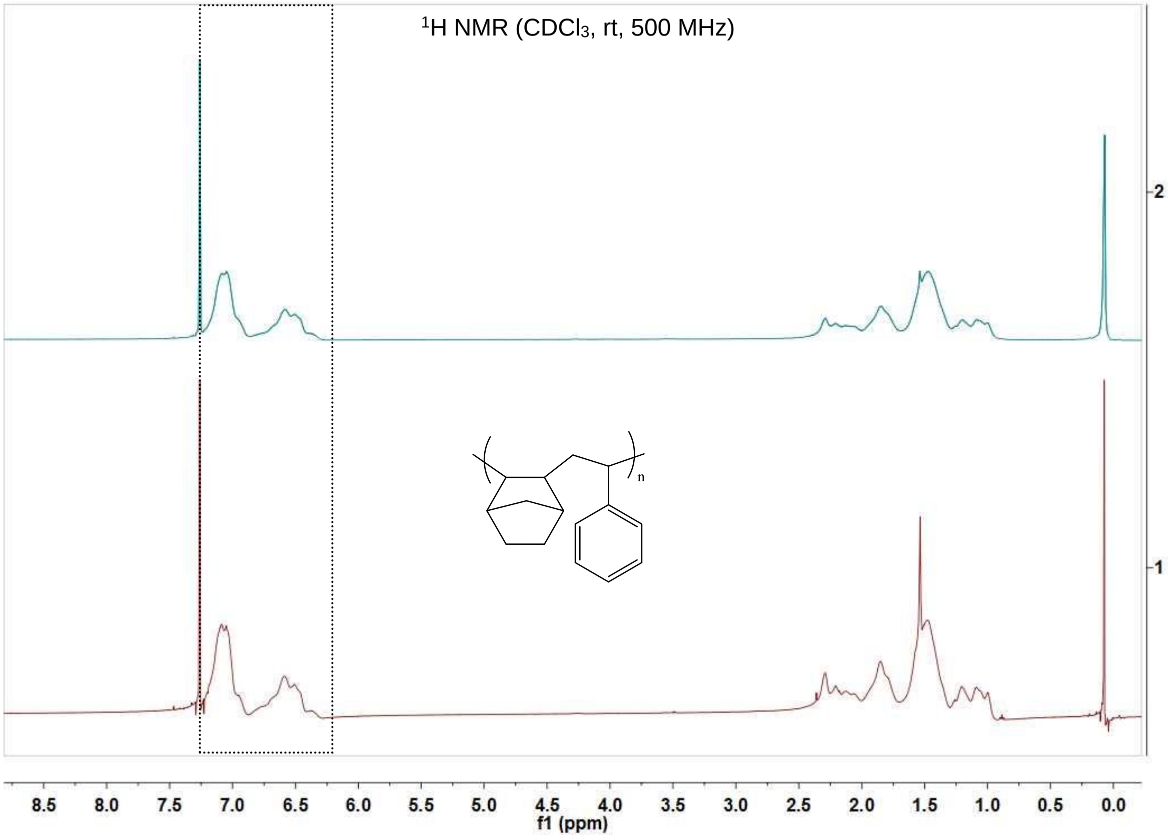

Figure S9. ^{1}H NMR spectra of N/S copolymers obtained by (**1**) Run 31 and (**2**) Run 34 [$CDCl_3$, 500 MHz].

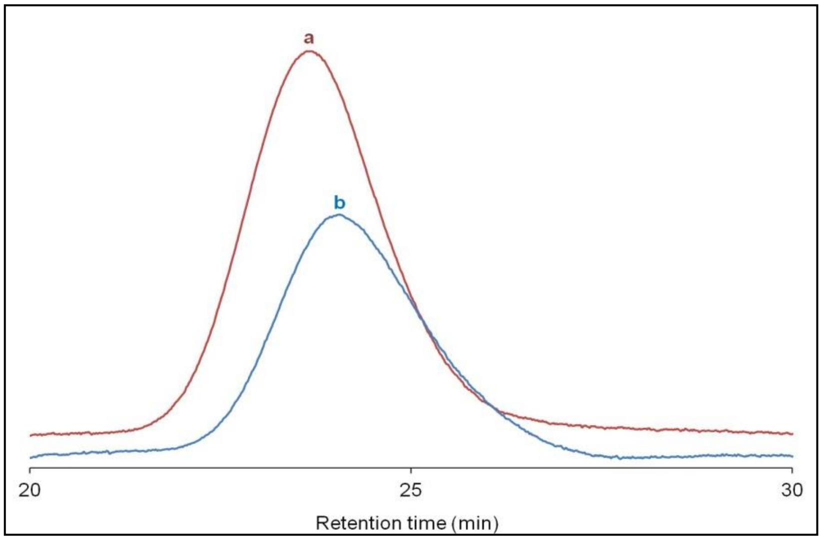

Figure S10. GPC traces N/S copolymers: **a**, Run 30; **b**, Run 33.

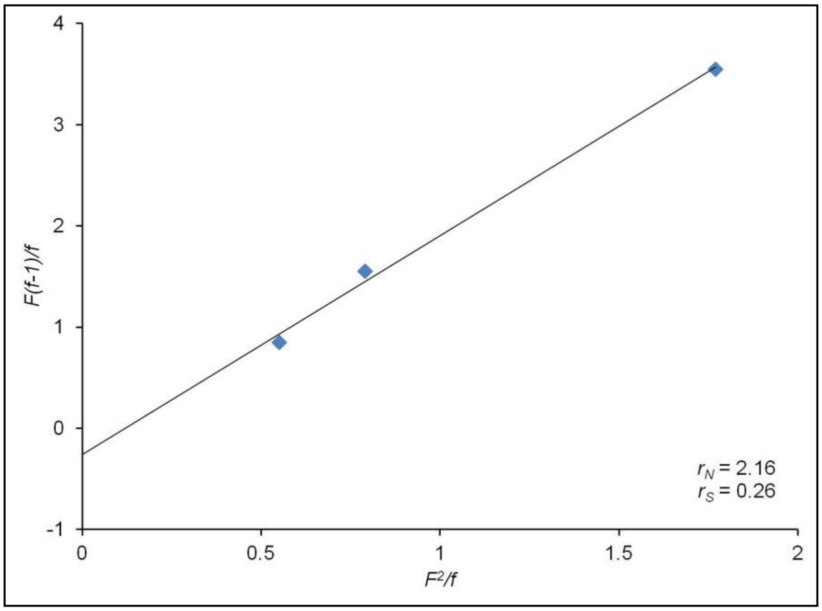

Fig. S11. Fineman-Ross plot for N/S copolymerization by **1c**-MMAO.

Table S1. Effects of monomer ratio of N/S copolymerization by **1b**-$B(C_6F_5)_3$

Run	(N/S)[a] (mmol/mmol)	Yield (g)	Activity[a]	f_S[b] (mol%)	M_n[c] (10^3)	M_w/M_n[c]	T_g[d]
29	40/10	0.088	18	18	38	2.0	251
30	40/20	0.110	22	27	33	1.7	197
31	40/30	0.133	27	33	30	1.7	175

Copolymerization conditions: Ni = 5 μmol, B/Ni = 4 (molar ratio), toluene (total volume 25 mL), temperature = 70 °C, time = 1 h. [a] Activity = $kg_{(polymer)}mol_{(Ni)}^{-1}h^{-1}$. [b] f_S are the content of S in the N/S copolymer determining by ^1H NMR spectrum. [c] Determined by GPC. [d] Determined by DSC.

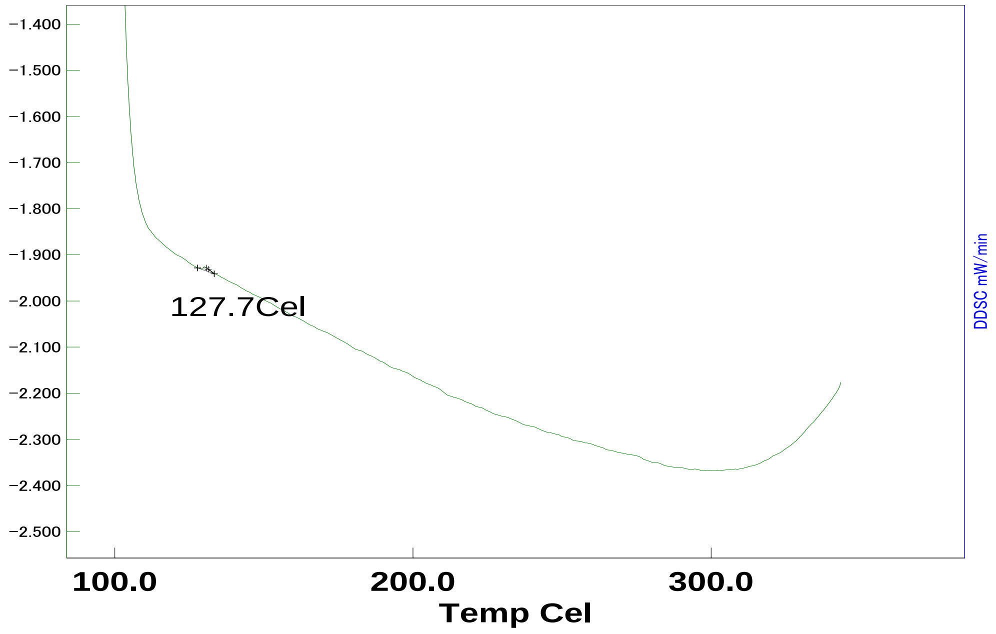

Figure S12. DSC curve of N/S copolymer (Run 31).

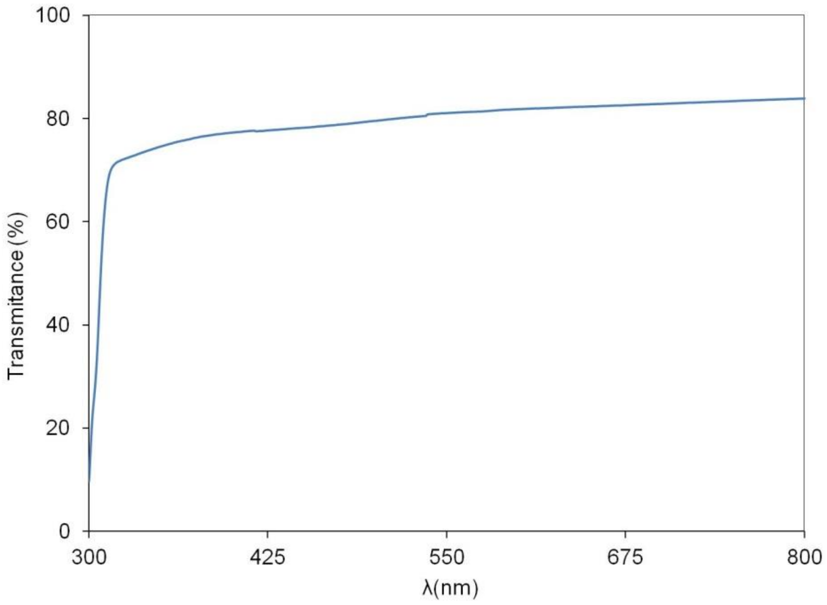

Figure S13. Transmittance of N/S copolymer thin film obtained by Run 35.

Reference:

(1) Hidai, M.; kashiwagi, T.; Ikeuchi, T.; Uchida, Y.; Oxidative Additions to Nickel (0): Preparation and Properties of a new Series of Arylnickel(ll) Complexes. *J.Organometal. Chem.* **1971**, *30*, 279-282.

(39) Okada, M.; Nakayama, Y.; Ikeda, T.; Shiono, T.; Synthesis of Uniquely Branched Polyethylene by Anilinonaphthoquinone Ligated Nickel Complex Activated with Tris(pentafluorophenyl)borane. *Macromol. Rapid Commun*. **2006**, *27,* 1418-1423.
